# Interleukin-33 modulates immune responses in cutaneous melanoma in a context-specific way

**DOI:** 10.18632/aging.202531

**Published:** 2021-02-17

**Authors:** Liang Peng, Wei Sun, Fanqin Wei, Lin Chen, Weiping Wen

**Affiliations:** 1 Department of Otorhinolaryngology Head and Neck Surgery, The First Affiliated Hospital, Sun Yat-sen University, Guangzhou 510080, Guangdong, China; 2 Institute of Otorhinolaryngology Head and Neck Surgery, Sun Yat-sen University, Guangzhou 510080, Guangdong, China; 3 Department of Otorhinolaryngology, The Sixth Affiliated Hospital, Sun Yat-sen University, Guangzhou 510655, Guangdong, China

**Keywords:** interleukin-33, cutaneous melanoma, tumor microenvironment, anti-tumor immunity

## Abstract

Controversial roles of interleukin-33 (IL-33) have been reported in melanoma from animal studies. We aimed to investigate the role of IL-33 in human cutaneous melanoma. RNA-seq data of 471 cases of cutaneous melanoma were retrieved from The Cancer Genome Atlas. The tumor microenvironment (TME) was deconstructed by the xCell algorithm using RNA-seq data. We evaluated the prognostic value of IL-33 and the relationship between IL-33 and immune components in TME. We also inferred the potential cellular sources of IL-33. All the analyses were conducted separately in three sub-cohorts, which are based on the biopsy sites of samples: primary melanoma; lymph node (LN) metastases; other metastases, including metastases to skin/soft tissue, or visceral sites. In the two metastasis sub-cohorts, IL-33 is associated with better prognosis and more active immune responses in the tumor. However, IL-33 is not a prognostic factor in the primary melanoma sub-cohort. Furthermore, we found that IL-33 is mainly derived from stromal cells in the metastasis sub-cohorts, and from epithelial cells/keratinocytes in the primary melanoma sub-cohort. These findings provide evidence for the context-specific anti-tumor effects of IL-33 in melanoma. And the distinct effects of IL-33 may be determined by the cellular sources of IL-33.

## INTRODUCTION

Cutaneous melanoma is one of the most aggressive skin cancers, accounting for more than 80% of skin-cancer related death [1]. Worldwide, 287,723 new cases of cutaneous melanoma and 60,712 deaths were registered in 2018 [2]. Most patients with early-staged melanoma can be cured by surgery [3]. However, effective treatments for patients with unresectable or metastatic melanoma are limited. Thanks to the improved understanding of the features and drivers of melanoma, therapeutic agents targeting the mitogen-activated protein kinase pathway (e.g., BRAF inhibitors, MEK inhibitors) have been approved for patients with *BRAF*-mutated melanoma [4]. Although targeted therapy exhibits good response rates, most patients will eventually develop resistance [5]. One of the promising immunotherapies, namely the immune checkpoint inhibitors, can achieve long-term remission or even curation through unleashing the host anti-tumor immune responses. However, there are still many melanoma patients who cannot benefit from immune checkpoint inhibitors [4]. Thus, additional effective therapeutic strategies for melanoma are required.

Interleukin-33 (IL-33), a member of the IL-1 family, is a multifunctional cytokine that participates in various inflammatory and autoimmune diseases with pathological or protectives roles [6, 7]. IL-33 is constitutively expressed at high levels in the nuclei of various cell types, including endothelial, epithelial and fibroblast-like cells [7, 8]. IL-33 is released by necrotic or damaged cells and secreted into the extracellular space, where it can bind to a heterodimer formed by orphan receptor ST2 and IL-1 receptor accessory protein, acting as an alarmin for tissue damage and infection [8]. The role of IL-33 in melanoma has been investigated in mouse models extensively, but only to draw controversial conclusions. Gao et al. reported that IL-33 overexpressed in transgenic mice could inhibit melanoma lung metastases through activating CD8^+^ T cells and natural killer (NK) cells [9]. Another study found that induced tumoral expression of IL-33 could promote anti-melanoma immune responses through interferon-γ (IFN-γ) producing CD8^+^ T cells and NK cells [10]. The anti-melanoma effects of exogenous IL-33 could also be mediated by eosinophils and dendritic cells (DCs) [11–13]. Jevtovic et al. found that systemically applied IL-33 could restrict primary melanoma growth, while the intranasally applied IL-33 promoted the growth of melanoma lung metastases through reducing the cytotoxicity of CD8^+^ T cells and enhancing regulatory T (Treg) cells [14]. Schuijs et al. found that intranasally administration of low-dose IL-33 could activate group 2 innate lymphoid cells (ILC2s) which orchestrated suppression of NK cell-mediated innate anti-tumor immunity, leading to increased melanoma lung metastases [15]. Long et al. also reported the contribution of IL-33-induced ILC2 to melanoma growth by weakening NK cell activation and tumor killing [16]. These controversial results may be due to the timing and dosage of IL-33 administration, and the context-specificity of IL-33’s effects. The role of IL-33 in melanoma still needs to be further explored.

With the advent of next-generation sequencing, huge amounts of cancer genomics data have been made publicly available. For example, The Cancer Genome Atlas (TCGA) program molecularly characterized a wide spectrum of cancer types, which greatly deepened our understanding of the genomic features of human cancers. Using the RNA-sequencing (RNA-seq) data of the cutaneous melanoma samples, we aim to dissect the tumor microenvironment (TME) and explore its relationship with IL-33 in these samples, hoping to find some new clues about IL-33’s effects on melanoma and hence to provide some therapeutic implications.

## RESULTS

### Included cases and IL-33 expression

A total of 471 cutaneous melanoma cases from TCGA were included in this study. According to the biopsy sites of the samples used for molecular analyses, the whole cohort was classified into 3 sub-cohorts: 103 samples were from primary cutaneous melanoma; 221 samples were from lymph node (LN) metastases; 147 samples were from other metastases, including metastases to skin/soft tissue, or visceral sites. Considering that the effects of IL-33 are highly dependent on the tissue environment, we conducted analyses in the three sub-cohorts separately.

**Figure 1:**
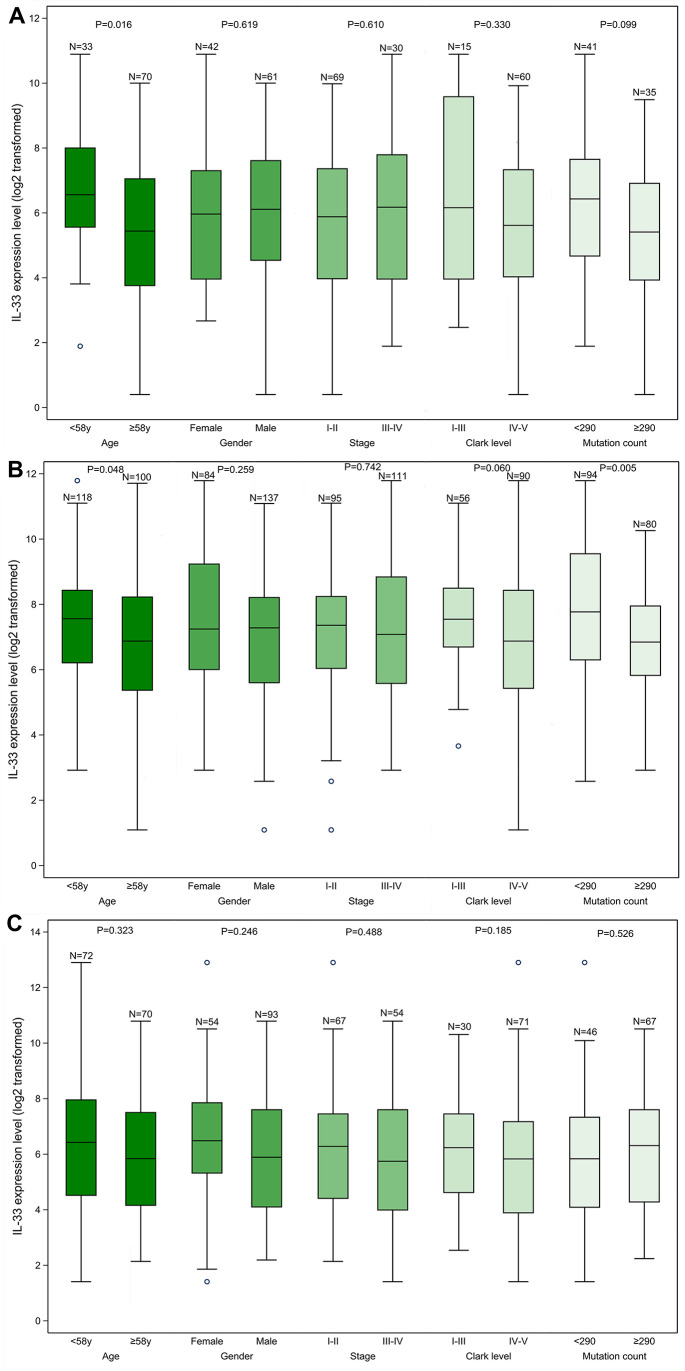
**The IL-33 expression level in clinicopathological subgroups.** (**A**) primary melanoma sub-cohort; (**B**) lymph node metastasis sub-cohort; (**C**) other metastasis sub-cohort. Boxplots represent values within the interquartile range (IQR) (boxes) and 1.5 × IQR (whiskers). Outliers are plotted as values > 1.5 × IQR (circles). P-values were calculated by the Mann-Whitney U test.

We found that samples from the LN metastasis sub-cohort have significantly higher IL-33 expression level compared with samples from the other two sub-cohorts (Supplementary Table 1). Within each sub-cohort, we compared the IL-33 expression level between subgroups defined by clinicopathological characteristics (Figure 1). The median values of age and mutation count of the whole cohort were chosen as cut-off values. In the primary melanoma sub-cohort, the expression level of IL-33 in samples from younger patients is higher than that in samples from elder patients (Figure 1A). In the LN metastasis sub-cohort, samples with lower mutation count or from younger patients have higher expression level of IL-33 (Figure 1B). However, in the other metastasis sub-cohort, no significant differences in the IL-33 expression level were found between subgroups (Figure 1C).

### Prognostic value of IL-33

**Figure 2:**
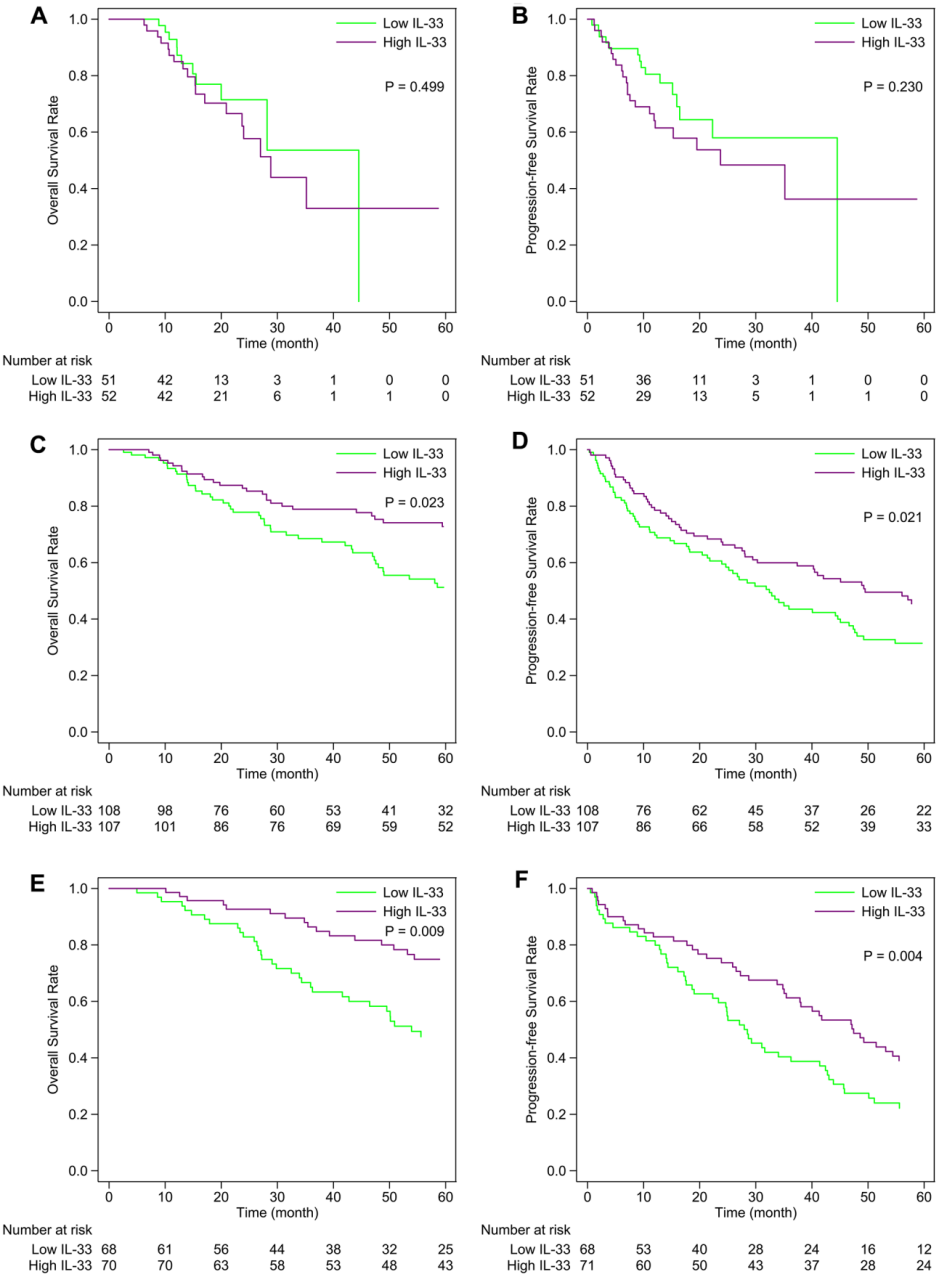
**Kaplan-Meier curves of the high and low IL-33 groups within each sub-cohort.** (**A**, **B**) primary melanoma sub-cohort; (**C**, **D**) lymph node metastasis sub-cohort; (**E**, **F**) other metastasis sub-cohort. P-values were calculated by the log-rank test.

In the primary melanoma sub-cohort, the expression level of IL-33 has no prognostic value in terms of overall survival (OS) or progression-free survival (PFS) (Figure 2A, 2B). However, the high expression level of IL-33 is associated with favorable OS and PFS in the LN (Figure 2C, 2D) and other metastasis sub-cohorts (Figure 2E, 2F). The prognostic values of IL-33 remain the same in the multivariate Cox regression model incorporating age as a covariate (data not shown).

### IL-33 and immune cells in TME

We compared the abundance of 34 types of immune cells in the tumor samples between the high and low IL-33 groups (Supplementary Table 3). Only three types of immune cells accumulate differently between the high and low IL-33 groups in the primary melanoma sub-cohort, with type 1 T helper (Th1) cells and natural killer T (NKT) cells more abundant in samples from the low IL-33 group, and conventional DCs more abundant in samples from the high IL-33 group. In the LN metastasis sub-cohort, 24 types of immune cells, including B cells, CD4^+^ T cells, CD8^+^ T cells, Th2 cells, NK cells, M1 macrophages, DCs et al., are more abundant in samples from the high IL-33 group; while three types of immune cells, including Th1 cells, NKT cells and basophils, are more abundant in samples from the low IL-33 group. In the other metastasis sub-cohort, 16 types of immune cells including B cells, CD4^+^ T cells, CD8+ T cells, NK cells, M1 macrophages, DCs et al., are more abundant in samples from the high IL-33 group; while Th1 cells and NKT cells are more abundant in samples from the low IL-33 group.

**Figure 3:**
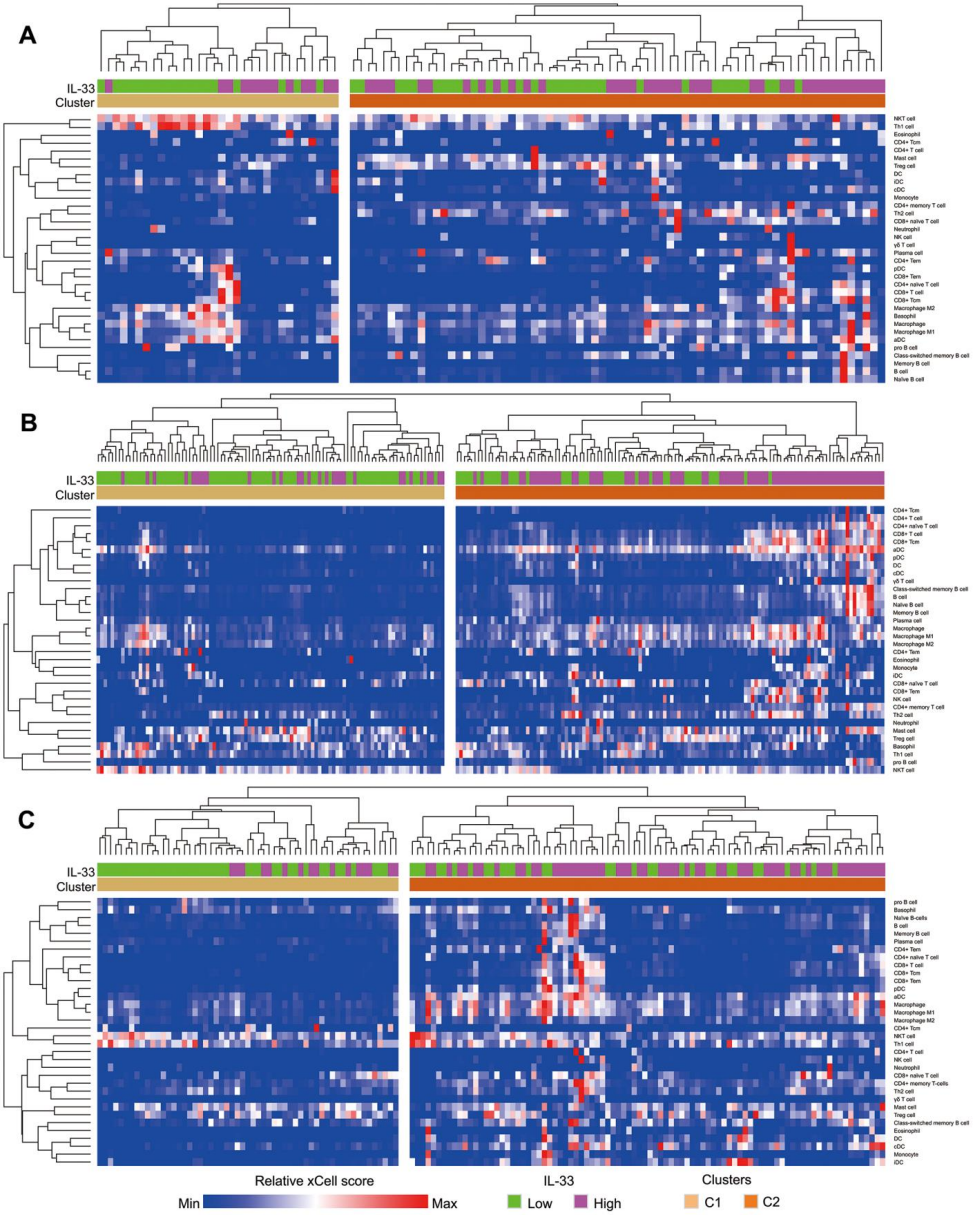
**Heatmap depicting the enrichment scores of immune cells in samples.** (**A**) primary melanoma sub-cohort; (**B**) lymph node metastasis sub-cohort; (**C**) other metastasis sub-cohort. Immune clusters were determined using unsupervised hierarchical clustering.

Based on xCell scores of the 34 types of immune cells, each sub-cohort was divided into 2 clusters. As is shown by the heatmap (Figure 3), samples of cluster 2 have more infiltrations of immunes cells compared with those of cluster 1. We also found that cluster 2 is significantly associated with the high IL-33 expression in the LN and other metastasis sub-cohorts (both of the Pearson’s χ^2^ test p-values < 0.001). However, the IL-33 expression level is not associated with immune clusters in the primary melanoma sub-cohort (Pearson’s χ^2^ test p-value = 0.179).

### IL-33 and differentially expressed genes

**Figure 4:**
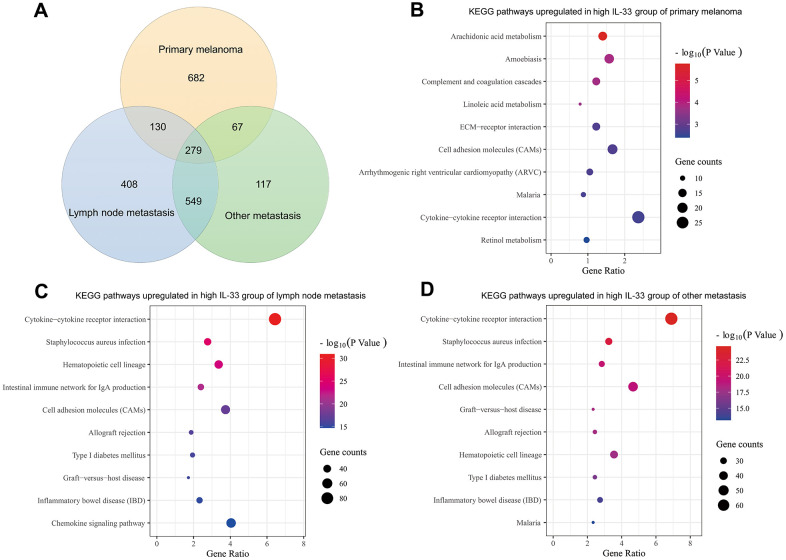
**Differentially expressed genes and enriched pathways.** (**A**) Venn diagram depicting genes expressed at higher levels in the high IL-33 groups; bubble plots depicting upregulated KEGG pathways in the high IL-33 groups of the primary melanoma sub-cohort (**B**), the LN metastasis sub-cohort (**C**), and the other metastasis sub-cohort (**D**).

Genes differentially expressed between the high and low IL-33 groups were extracted on the cBioPortal platform with the criteria of log ratio ≥ 1.0 and q-value < 0.05. Excluding IL-33, we found that 1158, 1366, and 1012 genes are expressed at higher levels in the high IL-33 group than in the low IL-33 group in the primary melanoma, LN metastasis, and other metastasis sub-cohorts, respectively (Figure 4A). These differentially expressed genes were subjected to Kyoto Encyclopedia of Genes and Genomes (KEGG) pathway enrichment analysis with the DAVID platform. We plotted the 10 enriched pathways with the lowest p-values (Figure 4B–4D). Consistent with analyses of immune cells abundance, pathways enriched in the high IL-33 group of the LN and other metastasis sub-cohorts are mostly related to the process of immune response and inflammation. However, pathways enriched in the high IL-33 group of the primary melanoma sub-cohort are less associated with the immune response.

### Potential cellular sources of IL-33

**Figure 5:**
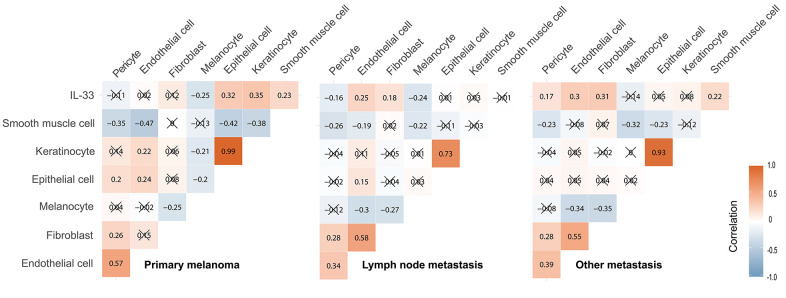
**Correlation between the IL-33 expression level and the abundance of potential cellular sources.** The Pearson correlation coefficients were shown in squares. Cross symbol means that the correlation is statistically insignificant.

Considering that the heterogeneity of IL-33’s effects may be due to the different cellular sources of IL-33 in the TME [17, 18], we tried to determine the potential sources of IL-33. In the TME, the potential sources of IL-33 are epithelial cells, keratinocytes, endothelial cells, pericytes, fibroblasts, smooth muscle cells, and tumor cells (namely, melanocytes). We investigated the correlation between the expression level of IL-33 and the abundance of these potential sources using Pearson’s correlation test (Figure 5). In the primary melanoma sub-cohort, the IL-33 expression level is positively correlated with the abundance of epithelial cells, keratinocyte, and smooth muscle cells. In the LN metastasis sub-cohort, the IL-33 expression level is positively correlated with the abundance of endothelial cells and fibroblasts. In the other metastasis sub-cohort, the IL-33 expression level is positively correlated with the abundance of endothelial cells, fibroblasts, pericytes, and smooth muscle cells. We also found that the abundance of these non-immune cells differs among sub-cohorts (Supplementary Table 2), which may explain why IL-33 is correlated with different cell populations in different sub-cohorts.

## DISCUSSION

We investigated the role of IL-33 in human cutaneous melanoma using TCGA datasets and found that the effects of IL-33 are dependent on the tumor context. The expression level of IL-33 in primary melanoma samples is less correlated to the immune cell components in TME and has no prognostic value in patients. On the contrary, the high expression level of IL-33 in the metastatic samples is associated with prolonged OS and PFS, which may be due to IL-33’s role in the anti-tumor immune responses. The differences of pathways enriched in the high IL-33 group between the primary melanoma and the metastasis sub-cohorts also reveal the context-specificity of IL-33’s effects.

In the primary melanoma sub-cohort, comparisons of immune cell components between the high and low IL-33 tumor samples reveal minor differences, indicating that IL-33 may not exert an immune modulation role in this setting. This is further supported by the pathway enrichment analysis of differentially expressed genes. In the metastasis sub-cohorts, the high expression of IL-33 is associated with more infiltrations of CD8^+^ T cells, NK cells and DCs in tumor samples, and these immune components have been proven to be important mediators of the anti-tumor effects of IL-33 in mouse melanoma models [9–11]. The abundance of eosinophils, another mediator of IL-33 [12, 13], is significantly higher in the high IL-33 group. However, the absolute abundance of eosinophils is very low in these tumor samples (Supplementary Table 3). To what extent the eosinophils play a role in the IL-33 induced anti-melanoma responses should be further determined. B cells, CD4^+^ T cells and M1 macrophages also accumulate more in the high IL-33 tumors than in the low IL-33 tumors, which may partially explain the better prognosis of the high IL-33 groups [19–23]. Notably, Th1 cells are more abundant in the low IL-33 samples rather than in the high IL-33 samples, which may be due to the enhanced Th2 polarization in the high IL-33 environment [24]. However, considering that Th1 cells and NKT cells are downregulated in the high IL-33 tumor samples in both the primary and metastatic settings, IL-33 itself may not account for the downregulation of Th1 cells and NKT cells.

Although the LN metastasis sub-cohort and the other metastasis sub-cohort were analyzed separately, the roles of IL-33 in the two settings are similar. Compared to the LN metastasis sub-cohort, the smaller number of cases in the other metastasis sub-cohort may lead to reduction in the power to detect differences in the abundance of immune cell components and expression level of genes between the high and low IL-33 groups. However, most of the differentially regulated immune cells in the other metastasis sub-cohort are also been regulated in the same direction in the LN metastasis sub-cohort; and the enriched KEGG pathways in the high IL-33 group of these two sub-cohorts are mostly overlapped.

The context-specific effects of IL-33 may be due to the different cellular sources of IL-33 in the tissue [17, 18]. Ectopic expression of IL-33 in melanoma cells can induce effective anti-tumor immune responses [10]. However, the expression level of IL-33 in human melanoma samples is uncorrelated or even negatively correlated to the abundance of melanocytes (Figure 5), indicating that melanoma cells may not be the source of IL-33. In the primary melanoma sub-cohort, the expression level of IL-33 is positively correlated with the abundance of epithelial cells, keratinocytes, and smooth muscle cells in the samples, indicating that IL-33 may mainly derive from these cells. Considering that epithelial cells and keratinocytes probably represent the adjacent normal epidermis which is involved in the biopsy samples, IL-33 expressed in these cells may not be released into the extracellular space or interact with the TME. This may explain why IL-33 does not have effects on immune modulation or prognostic values in the primary melanoma sub-cohort. In both the LN and the other metastasis sub-cohorts, the expression level of IL-33 is positively correlated with the abundance of endothelial cells and fibroblasts, indicating that these two types of cells may be the main sources of IL-33. Endothelial cells and fibroblasts are important components of tumor stroma, and they actively interact with other TME components and tumor cells [25]. It is more likely that IL-33 released from these stromal cells could interact with the TME and enhance the anti-tumor immune responses, which may explain the better prognosis of the high IL-33 groups of the metastasis sub-cohorts.

Several limitations of this study should be noted. First, the immune cell components scored using xCell were not validated using traditional tumor imaging methods, such as immunohistochemistry. We were not able to discriminate the immune cells in each sample that deeply infiltrated in the tumor from those that were adjacent to tumor or physiologically present due to involvement of lymphatic tissue in the sample. Second, our findings are derived from TCGA database and hence should be validated by real-world data in the future. Third, this is a cross-sectional study, and only correlation could be deduced. The causal relationship between IL-33 and the prognosis and immune infiltrations should be further validated by well-designed *in vitro/vivo* experiments.

In conclusion, we found that IL-33 has a context-specific role in human cutaneous melanoma, which may be determined by the cellular sources of IL-33. In primary melanoma samples, IL-33 is probably derived from the epithelial tissue and is not associated with prognosis. In metastatic melanoma samples, IL-33 is probably derived from stromal cells and may improve prognosis through enhancing anti-tumor immune responses. This study helps us understand the role of IL-33 in cutaneous melanoma and provides possible therapeutic implications of targeting this cytokine.

## MATERIALS AND METHODS

### Dataset

A total of 471 cutaneous melanoma cases from TCGA were included in this study. The RNA-seq and mutation burden data of melanoma samples and the clinical information of patients were obtained from the cBioPortal for Cancer Genomics in June 2020 (http://www.cbioportal.org) [26]. Gene expression values were presented as RNA-Seq by Expectation Maximization (RSEM) data normalized to the upper quartile of total reads within each sample [27].

As this study was conducted using publicly available data, ethics approval and informed consent were not required.

### TME decomposition

The bulk RNA-seq data of samples were used to dissect the TME using xCell algorithm, which is available at http://xCell.ucsf.edu/ [28]. Enrichment scores for 64 cell types, including immune cells and stromal cells, were calculated to represent the abundance of each cell component within the sample.

### Statistical analyses

The expression level of IL-33 was compared using the Mann-Whitney U test or Kruskal-Wallis test. We divided patients into the high IL-33 group and the low IL-33 group based on the median IL-33 expression level within each sub-cohort. The OS and PFS were compared between the high and low IL-33 groups using the log-rank test. To evaluate the effects of IL-33 on tumor immune infiltrations, xCell scores of 34 types of immune cells were compared between the high and low IL-33 groups within each sub-cohort using the Mann-Whitney U test, and q-values were calculated to account for multiple testing. Based on enrichment scores of immune cells, unsupervised hierarchical clustering was performed to divide each sub-cohort into two immune clusters. The relationship between the expression level of IL-33 and the immune cluster was evaluated using Pearson’s χ^2^ test. The correlations between the IL-33 expression level and the abundance of the potential cellular sources of IL-33 (epithelial cells, keratinocytes, endothelial cells, pericytes, fibroblasts, smooth muscle cells, and melanocytes represented by xCell scores) were evaluated by the Pearson’s correlation test. Differential gene expression analysis between the high IL-33 group versus the low IL-33 group was performed with the cBioPortal platform. KEGG pathway enrichment analysis of differentially expressed genes was performed with the DAVID platform (version 6.8, https://david.ncifcrf.gov) [29]. Two-tailed p-values or q-values < 0.05 were considered statistically significant.

## Supplementary Material

Supplementary Tables 1 and 2

Supplementary Table 3
