# Supplementary material for: Interleukin-33 modulates immune responses in cutaneous melanoma in a context-specific way
Source: Aging (Albany NY). 2021 Feb 17;13(5):6740–51. doi: 10.18632/aging.202531 (PMC7993738; doi:10.18632/aging.202531)
Supplement: Supplementary Tables 1 and 2 [file aging-13-202531-s001.pdf]

SUPPLEMENTARY TABLES

Supplementary Table 1. The expression level of IL-33 among sub-cohorts.

	Primary melanoma sub-cohort	LN metastasis sub-cohort	Other metastasis sub-cohort	p-value*
**IL-33 expression level (log2 transformed)**	6.05 (3.97-7.50)	7.28 (5.83-8.52)	6.17 (4.25-7.84)	< 0.001

Data presented as median (interquartile range).
Abbreviation: IL-33, interleukin-33; LN, lymph node.
*Kruskal-Wallis test. Pairwise comparisons: primary melanoma sub-cohort vs. LN metastasis sub-cohort (p <0.001); primary melanoma sub-cohort vs. other metastasis sub-cohort (p = 0.588); LN metastasis sub-cohort vs. other metastasis sub-cohort (p <0.001).

Supplementary Table 2. The abundance of non-immune cells among sub-cohorts.

Cell types	Primary melanoma sub-cohort	LN metastasis sub-cohort	Other metastasis sub-cohort	p-value*
**Melanocyte**	0.241 (0.187-0.279)	0.212 (0.132-0.266)	0.192 (0.109-0.266)	< 0.001
**Epithelial cell**	0.023 (0.003-0.140)	0.003 (0-0.007)	0.003 (0-0.008)	< 0.001
**Keratinocyte**	0.006 (0-0.063)	0 (0-0)	0 (0-0)	< 0.001
**Fibroblast**†	0 (0-0)	0 (0-0)	0 (0-0)	0.011
**Endothelial cell**	0.001(0-0.020)	0.015 (0-0.034)	0.014 (0.001-0.036)	< 0.001
**Pericyte**	0 (0-0.003)	0 (0-0.006)	0 (0-0.022)	0.035
**Smooth muscle cell**	0.298 (0.198-0.383)	0.326 (0.257-0.393)	0.357 (0.267-0.407)	0.006

Data presented as median (interquartile range).
Abbreviation: IL-33, interleukin-33; LN, lymph node.
*Kruskal-Wallis test.
†The mean xCell scores of fibroblasts are 0.001, 0.009, and 0.009 for the primary melanoma, LN metastasis, and other metastasis sub-cohorts, respectively.
