# Supplementary material for: Interleukin-33 modulates immune responses in cutaneous melanoma in a context-specific way
Source: Aging (Albany NY). 2021 Feb 17;13(5):6740–51. doi: 10.18632/aging.202531 (PMC7993738; doi:10.18632/aging.202531)
Supplement: Supplementary Table 3 [file aging-13-202531-s002.docx]

**Supplementary Table 3. The comparison of xCell scores of immune cells between the high and low IL-33 groups within each sub-cohort.**

| **Immune cells** | **Primary melanoma sub-cohort** | | | **Lymph node metastasis sub-cohort** | | | **Other metastasis sub-cohort** | | |
| --- | --- | --- | --- | --- | --- | --- | --- | --- | --- |
|  | Low IL-33 | High IL-33 | q-value^*^ | Low IL-33 | High IL-33 | q-value^*^ | Low IL-33 | High IL-33 | q-value^*^ |
|  | N = 51 | N = 52 |  | N = 110 | N = 111 |  | N = 73 | N = 74 |  |
| B cell | 0.017 (0.007-0.025) | 0.009 (0-0.030) | 0.630 | 0.040 (0.014-0.090) | 0.116 (0.053-0.244) | **< 0.001** | 0.016 (0.004-0.033) | 0.033 (0.010-0.092) | **0.011** |
| Class-switched memory B cell | 0.006 (0-0.011) | 0.002 (0.007-0.021) | 0.351 | 0.016 (0.006-0.030) | 0.033 (0.018-0.064) | **< 0.001** | 0.010 (0.001-0.023) | 0.014 (0.006-0.031) | 0.059 |
| Memory B cell | 0 (0-0) | 0 (0-0.002) | 0.583 | 0 (0-0.011) | 0.020 (0-0.065) | **< 0.001** | 0 (0-0.002) | 0 (0-0.007) | 0.426 |
| Naïve B cell | 0.001 (0-0.005) | 0 (0-0.005) | 0.697 | 0.006 (0-0.013) | 0.021 (0.007-0.039) | **< 0.001** | 0.002 (0-0.005) | 0.004 (0-0.014) | **0.032** |
| Pro-B cell | 0 (0-0) | 0 (0-0) | 0.412 | 0 (0-0.007) | 0 (0-0.008) | 0.138 | 0 (0-0.002) | 0 (0-0.002) | 0.672 |
| Plasma cell | 0.005 (0.003-0.008) | 0.003 (0-0.009) | 0.473 | 0.008 (0.004-0.015) | 0.014 (0.007-0.023) | **< 0.001** | 0.006 (0.002-0.010) | 0.006 (0.002-0.011) | 0.869 |
| CD4^+^ T cell | 0 (0-0) | 0 (0-0) | 0.351 | 0 (0-0) | 0 (0-0.001) | **< 0.001** | 0 (0-0) | 0 (0-0) | 0.204 |
| CD4^+^ naïve T cell | 0 (0-0) | 0 (0-0.004) | 0.351 | 0 (0-0.006) | 0.021 (0-0.066) | **< 0.001** | 0 (0-0) | 0 (0-0.015) | **< 0.001** |
| CD4^+^ memory T cell | 0.010 (0-0.018) | 0.014 (0.008-0.022) | 0.266 | 0.020 (0.011-0.034) | 0.047 (0.026-0.070) | **< 0.001** | 0.018 (0.007-0.025) | 0.027 (0.015-0.045) | **< 0.001** |
| CD4^+^ Tcm | 0 (0-0) | 0 (0-0.014) | 0.161 | 0 (0-0) | 0 (0-0) | 0.342 | 0 (0-0) | 0 (0-0) | 0.979 |
| CD4^+^ Tem | 0 (0-0.006) | 0 (0-0.013) | 0.310 | 0 (0-0.004) | 0 (0-0.014) | **0.033** | 0 (0-0.004) | 0 (0-0.020) | **0.032** |
| CD8^+^ T cell | 0 (0-0.003) | 0.002 (0-0.020) | 0.153 | 0 (0-0.018) | 0.037 (0.007-0.081) | **< 0.001** | 0 (0-0) | 0.006 (0-0.038) | **< 0.001** |
| CD8^+^ naïve T cell | 0 (0-0.007) | 0.005 (0-0.010) | 0.153 | 0.003 (0-0.008) | 0.003 (0-0.010) | 0.705 | 0 (0-0.003) | 0.003 (0-0.013) | **< 0.001** |
| CD8^+^ Tcm | 0 (0-0.021) | 0.002 (0-0.017) | 0.295 | 0.010 (0-0.044) | 0.074 (0.022-0.143) | **< 0.001** | 0 (0-0.013) | 0.019 (0-0.071) | **< 0.001** |
| CD8^+^ Tem | 0 (0-0) | 0 (0-0) | 0.726 | 0 (0-0) | 0 (0-0.016) | **< 0.001** | 0 (0-0) | 0 (0-0.002) | **0.006** |
| Th1 cell | 0.076 (0.044-0.116) | 0.040 (0.021-0.073) | **0.004** | 0.058 (0.033-0.107) | 0.032 (0.015-0.058) | **< 0.001** | 0.068 (0.035-0.111) | 0.041 (0.022-0.067) | **0.001** |
| Th2 cell | 0.013 (0-0.043) | 0.016 (0.001-0.053) | 0.680 | 0.029 (0.007-0.064) | 0.042 (0.021-0.087) | **0.013** | 0.023 (0.010-0.049) | 0.029 (0.007-0.070) | 0.235 |
| Treg cell | 0.013 (0-0.026) | 0.011 (0-0.023) | 0.672 | 0.018 (0-0.032) | 0.015 (0-0.030) | 0.705 | 0.013 (0-0.028) | 0.014 (0.003-0.031) | 0.672 |
| γδ T cell | 0 (0-0) | 0 (0-0) | 0.351 | 0 (0-0) | 0 (0-0) | **< 0.001** | 0 (0-0) | 0 (0-0) | **0.004** |
| NK cell | 0 (0-0) | 0 (0-0) | 0.819 | 0 (0-0) | 0 (0-0.004) | **0.013** | 0 (0-0) | 0 (0-0.001) | **0.001** |
| NKT cell | 0.145 (0.106-0.210) | 0.085 (0.062-0.128) | **< 0.001** | 0.115 (0.080-0.150) | 0.082 (0.058-0.115) | **< 0.001** | 0.121 (0.064-0.163) | 0.081 (0.054-0.113) | **0.012** |
| Neutrophil | 0 (0-0) | 0 (0-0) | 0.960 | 0 (0-0) | 0 (0-0) | 0.329 | 0 (0-0) | 0 (0-0) | 0.661 |
| Eosinophil | 0 (0-0) | 0 (0-0) | 0.351 | 0 (0-0) | 0 (0-0) | **0.022** | 0 (0-0) | 0 (0-0) | 0.054 |
| Basophil | 0.027 (0.011-0.057) | 0.019 (0-0.041) | 0.310 | 0.031 (0.016-0.055) | 0.027 (0.004-0.048) | **0.049** | 0.035 (0.015-0.056) | 0.029 (0.010-0.055) | 0.563 |
| Mast cell | 0.003 (0.001-0.007) | 0.006 (0.002-0.009) | 0.161 | 0.005 (0.001-0.009) | 0.006 (0.003-0.010) | 0.177 | 0.008 (0.003-0.011) | 0.006 (0.004-0.009) | 0.346 |
| Monocyte | 0 (0-0) | 0 (0-0) | 0.726 | 0 (0-0) | 0 (0-0.008) | **< 0.001** | 0 (0-0) | 0 (0-0.003) | 0.067 |
| Macrophage | 0.033 (0.020-0.046) | 0.027 (0.016-0.048) | 0.726 | 0.037 (0.020-0.062) | 0.059 (0.038-0.084) | **< 0.001** | 0.029 (0.013-0.067) | 0.046 (0.028-0.086) | **0.012** |
| Macrophage M1 | 0.017 (0.010-0.029) | 0.016 (0.009-0.028) | 0.933 | 0.022 (0.010-0.041) | 0.037 (0.024-0.059) | **< 0.001** | 0.017 (0.006-0.037) | 0.027 (0.016-0.054) | **0.012** |
| Macrophage M2 | 0.013 (0.005-0.022) | 0.011 (0.005-0.019) | 0.399 | 0.016 (0.009-0.027) | 0.019 (0.010-0.029) | 0.054 | 0.013 (0.006-0.024) | 0.017 (0.010-0.028) | 0.170 |
| DC | 0 (0-0.001) | 0 (0-0.002) | 0.529 | 0 (0-0.002) | 0.005 (0.001-0.013) | **< 0.001** | 0 (0-0) | 0.001 (0-0.006) | **< 0.001** |
| aDC | 0.051 (0.019-0.112) | 0.074 (0.027-0.142) | 0.351 | 0.091 (0.032-0.146) | 0.189 (0.126-0.298) | **< 0.001** | 0.047 (0.008-0.094) | 0.107 (0.053-0.196) | **< 0.001** |
| cDC | 0.004 (0-0.009) | 0.013 (0.005-0.029) | **< 0.001** | 0.007 (0-0.019) | 0.036 (0.018-0.074) | **< 0.001** | 0.006 (0-0.015) | 0.029 (0.010-0.047) | **< 0.001** |
| iDC | 0.014 (0-0.037) | 0.016 (0-0.033) | 0.853 | 0.009 (0-0.050) | 0.032 (0-0.105) | **0.033** | 0 (0-0.033) | 0.011 (0-0.067) | 0.096 |
| pDC | 0.007 (0.002-0.019) | 0.007 (0.001-0.017) | 0.917 | 0.008 (0.002-0.024) | 0.030 (0.009-0.071) | **< 0.001** | 0.004 (0.001-0.010) | 0.007 (0.001-0.034) | 0.054 |

Data presented as median (interquartile range).

Abbreviation: Tcm, central memory T cell; Tem, effector memory T cell; Th1 cell, type 1 T helper cell; Th2 cell, type 2 T helper cell; Treg cell, regulatory T cell; NK cell, natural killer cell; NKT cell, natural killer T cell; DC, dendritic cell; aDC, activated DC; cDC, conventional DC; iDC, immature DC; pDC, plasmacytoid DC.

^*^Mann-Whitney U-test.
